# Relations between vital capacity, CO diffusion capacity and computed tomographic findings of former asbestos-exposed patients: a cross-sectional study

**DOI:** 10.1186/s12995-020-00272-1

**Published:** 2020-07-01

**Authors:** Alexandra Marita Preisser, Katja Schlemmer, Robert Herold, Azien Laqmani, Claudia Terschüren, Volker Harth

**Affiliations:** 1 Institute for Occupational and Maritime Medicine (ZfAM), University Medical Center Hamburg-Eppendorf (UKE), Hamburg, Germany; 2 Department of Diagnostic and Interventional Radiology and Nuclear Medicine, University Medical Center Hamburg-Eppendorf (UKE), Hamburg, Germany

**Keywords:** Asbestosis, Pleural plaques, Lung function, CO diffusion capacity, Vital capacity, Parenchymal bands, Subpleural curvilinear lines, Round atelectasis, Thorax computed tomography, ICOERD

## Abstract

**Background:**

Asbestos-related lung diseases are one of the leading diagnoses of the recognized occupational diseases in Germany, both in terms of their number and their socio-economic costs. The aim of this study was to determine whether pulmonary function testing (spirometry and CO diffusion measurement (D_LCO_)) and computed tomography of the thorax (TCT) are relevant for the early detection of asbestos-related pleural and pulmonary fibrosis and the assessment of the functional deficiency.

**Methods:**

The records of 111 formerly asbestos-exposed workers who had been examined at the Institute for Occupational and Maritime Medicine, Hamburg, Germany, with data on spirometry, D_LCO_ and TCT were reviewed. Workers with substantial comorbidities (cardiac, malignant, silicosis) and/or pulmonary emphysema (pulmonary hyperinflation and/or TCT findings), which, like asbestosis, can lead to a diffusion disorder were excluded. The remaining data of 41 male workers (mean 69.8 years ±6.9) were evaluated. The TCT changes were coded according to the International Classification of High-resolution Computed Tomography for Occupational and Environmental Respiratory Diseases (ICOERD) by radiologists and ICOERD-scores for pleural and pulmonary changes were determined. Correlations (ρ), Cohens κ and accuracy were calculated.

**Results:**

In all 41 males the vital capacity (VC in % of the predicted value (% pred.)) showed only minor limitations (mean 96.5 ± 18.0%). The D_LCO_ (in % pred.) was slightly reduced (mean 76.4 ± 16.6%; median 80.1%); the alveolar volume related value (D_LCO_/VA) was within reference value (mean 102 ± 22%). In the TCT of 27 workers pleural asbestos-related findings were diagnosed whereof 24 were classified as pulmonary fibrosis (only one case with honey-combing). Statistical analysis provided low correlations of VC (*ρ* = − 0.12) and moderate correlations of D_LCO_ (− 0.25) with pleural plaque extension. The ICOERD-score for pulmonary fibrosis correlated low with VC (0.10) and moderate with D_LCO_ (− 0.23); D_LCO_ had the highest accuracy with 73.2% and Cohens κ with 0.45. D_LCO_/VA showed no correlations to the ICOERD-score. The newly developed score, which takes into account the diffuse pleural thickening, shows a moderate correlation with the D_LCO_ (*ρ* = − 0.35, *p* < 0.05).

**Conclusions:**

In formerly asbestos-exposed workers, lung function alterations and TCT findings correlated moderate, but significant using D_LCO_ and ICOERD-score considering parenchymal ligaments, subpleural curvilinear lines, round atelectases and pleural effusion in addition to pleural plaque extension. D_LCO_ also showed highest accuracy in regard to pulmonary findings. However, VC showed only weaker correlations although being well established for early detection. Besides TCT the determination of both lung function parameters (VC and D_LCO_) is mandatory for the early detection and assessment of functional deficiencies in workers formerly exposed to asbestos.

## Background

Occupational diseases due to asbestos exposure are the fourth most confirmed occupational disease in Germany in 2018 with 3401 cases [1]. In 2018, 65% of all deaths due to an occupational disease were caused by asbestos [2]. As a result of the widespread use of the building material asbestos and the long latency between exposure and first symptoms, a considerable number of unreported cases must be assumed. About 40% (€ 250 million) of the health care costs and compensatory payments covered by the German Social Accident Insurance are attributed to occupational diseases caused by asbestos [3].

In addition to malignant diseases (which are not discussed here), the consequences of exposure to asbestos are typical pleural plaques, pleuritis and pulmonary fibrosis (asbestosis) with the consequence of a restrictive ventilation disorder. Restrictive lung diseases show a reduction of all lung volumes at normal relative one-second capacity (FEV_1_/FVC) and a gas exchange disorder with decrease of the diffusion capacity for carbon monoxide (D_LCO_ and D_LCO_/VA).

The pathognomonic changes of the pleura and the typical changes of the lung parenchyma caused by asbestos are better identifiable on TCT than on chest X-ray [4]. The lung parenchyma signs include a centrilobular increase in density, intralobular structures that are not ordered in septa, and thickened interlobular septa. Subpleural curvilinear lines (SC) running parallel to the thoracic wall indicate the onset of fibrosis. So-called parenchymal bands (PB) are visible in TCT as pleuropulmonary fibrotic strands. Honeycombing is the term for maximum fibrosis, i.e. the destruction of the lung parenchyma [5–9]. Asbestosis is difficult to detect in early stages [9, 10].

So far, there are different views on the effect of plaques on lung function: Some studies show that a deterioration of lung function, e.g. as a restriction of vital capacity (VC) and forced expiratory vital capacity over 1 s (FEV_1_), can be detected in patients with proven exposure to asbestos and pulmonary fibrosis in X-ray or CT scans [11–13]; only a few publications also consider CO diffusion capacity (D_LCO_) [14–16]. According to our own studies, limitations in diffusion capacity are the most sensitive parameter for determining a disease with an asbestos-related cause [17]. Even though there is evidence in the studies mentioned above, it is still unclear whether lung function restrictions are in fact present, if, besides pleuroparietal plaques, no or only minimal changes (like SC or PB) caused by asbestos are visible in the CT, or whether structural alterations of the lung parenchyma are mandatory for lung function impairments. By this, secondary prevention measures for the detection and confirmation of compensatory payments should be initiated at an early stage. Non-invasive, low-radiation and economically appropriate methods like lung function test should be available for this purpose. So, the aim of this study was to determine whether pulmonary function testing (spirometry and D_LCO_) and computed tomography of the thorax (TCT) are relevant for the early detection of asbestos-related pleural and pulmonary fibrosis and the assessment of the functional deficiency.

## Methods

The study was conducted retrospectively with data from patient records of 111 occupationally asbestos-exposed workers and was obtained by experts from the Institute for Occupational and Maritime  Medicine (ZfAM), Hamburg (Germany), between January 2013 and October 2016 for clarification/confirmation or follow-up of an asbestos-related disease. In addition to TCT and medical history, following parameters of lung function were gathered: VC measurement (VC_max_, FVC), CO  diffusion capacity (D_LCO_, and D_LCO_/VA), spirometry (FEV_1_/FVC), body plethysmography, and if necessary bronchospasmolysis test to determine non-reversible pulmonary hyperinflation. Furthermore, haemoglobin value and percent of CO haemoglobin were determined. The medical history was screened for other diseases that restrict lung function and pulmonary gas exchange to exclude patients with e.g. lung cancer, silicosis, sarcoidosis or hypersensitivity pneumonitis as well as cardiac diseases. Moreover, patients with pulmonary emphysema were not included in the study, as reduced diffusion capacity may be the result of both asbestosis and emphysema. For the diagnosis of pulmonary emphysema, shown by 15 patients, at least one of the following criteria had to be met: (1) Increase of residual volume (RV) and ratio of residual volume to total lung capacity (RV/TLC > upper limit of normal (ULN)), not reversible after bronchospasmolysis [18]. (2) “Emphysema kink” in the flow-volume curve [19], (3) Radiological findings of an emphysema.

Finally, 41 male subjects met the quality criteria of completeness and comparability of the examination and assessment methods. Information on age, height and weight, duration of previous exposure to asbestos and smoking behaviour (former or active smoker; number of packyears (py)) were included. Every person who is examined in our outpatient clinic (ZfAM) gives their written consent that we may conduct further studies with their anonymized data and write publications about. According to the Ethics Committee of the Hamburg Medical Association an extra ethics vote is not necessary due to in-house research and retrospective evaluation of the data.

### Spirometry, body plethysmography and CO diffusion capacity

Spirometry and body plethysmography had been carried out according to the quality criteria of the European Respiratory Society (ERS) [20], the American Thoracic Society (ATS) [21] and German Guideline for standardization of spirometry [22], demanding three artefact-free spirometry breathing manoeuvres. Following the guidelines, the higher result of two reproducible manoeuvres was selected. For the interpretation of lung function, the reference values for spirometry of the Global Lung Initiative (GLI) [23] and for body plethysmography of the European Coal and Steel Community (ECSC) [24] were used. D_LCO_ was performed in single breath (SB) method according to the recommendations of MacIntyre et al. [25] and Graham et al. [26] using the predicted values by Cotes et al. [27]. If the haemoglobin (Hb) value was determined, the D_LCO_ values were adjusted (corr. D_LCO_  = D_LCO_*(10.22 + Hb)/(1.7*Hb) according to Mottram et al. [28]. This adjustment was performed in 22 of 41 cases; in cases where there was no clinical evidence of anemia and the Hb value was missing, an Hb value of 14.6 g/dl was assumed. All statistical analyses were performed with corr.  D_LCO_ values calculated to a normal Hb value.

### Computed tomography of the lung

To ensure a standardised evaluation, the International Classification of Occupational and Environmental Respiratory Disease (ICOERD) was used to assess the TCTs [29, 30]. The semi-quantitative description and coding were performed by radiologists following the guidelines for assessment and image data documentation by Hering et al. [8]. In 32 (78%) of the subjects, TCT was performed with a maximum interval of 30 days after/before administering the lung function test; in five subjects within 31 to 180 days and in four subjects within 181 to 365 days (mean 42 days, SD 82).

#### The coding scheme of the international classification of occupational and environmental respiratory diseases (ICOERD)

The ICOERD coding scheme is regarded as the current standard for the diagnosis of computed tomographic image material in pneumoconiosis [29]. In the section “Lung” the International Classification indicates irregular and/or linear compression and honey-combing. In the “Pleura” section, two forms of pleural changes can be differentiated: the parietal type with tableland-shaped or flat, partly spindle-shaped thickenings of the pleura without subpleural fibrosis and the visceral type with thickenings of the pleura with adjacent parenchymal ligaments. The parietal type can be regarded as highly asbestos-related pleural plaques, while the visceral type can be caused differently [8]. According to the Helsinki criteria, the minimum requirement for a clear diagnosis of asbestosis in the TCT is fibrosis, which is represented by at least two bilateral irregular densifications of the lower lung sections and/or bilateral honeycombing with a total of more than 2 points [10, 31].

#### Scores

ICOERD is used to assign scores to various structures, some of which are used as the basis for this evaluation. *Score A* quantifies the pleural plaques and thus reflects their local extent; according to the occurrence at the right (R) and left (L) pleura and there depending on the proportion with U, M and L (for upper, middle and lower field, respectively R and L) [5]. This score ranges between 0 and 6 points and mainly takes into account plaques of the parietal type. *Score B* for the occurrence of fibrosis of the lung parenchyma is derived from the ICOERD according to the total variance of irregular and/or linear opacities. The score/point value for pulmonary fibrosis can range from 0 to 18 points.

**Fig. 1:**
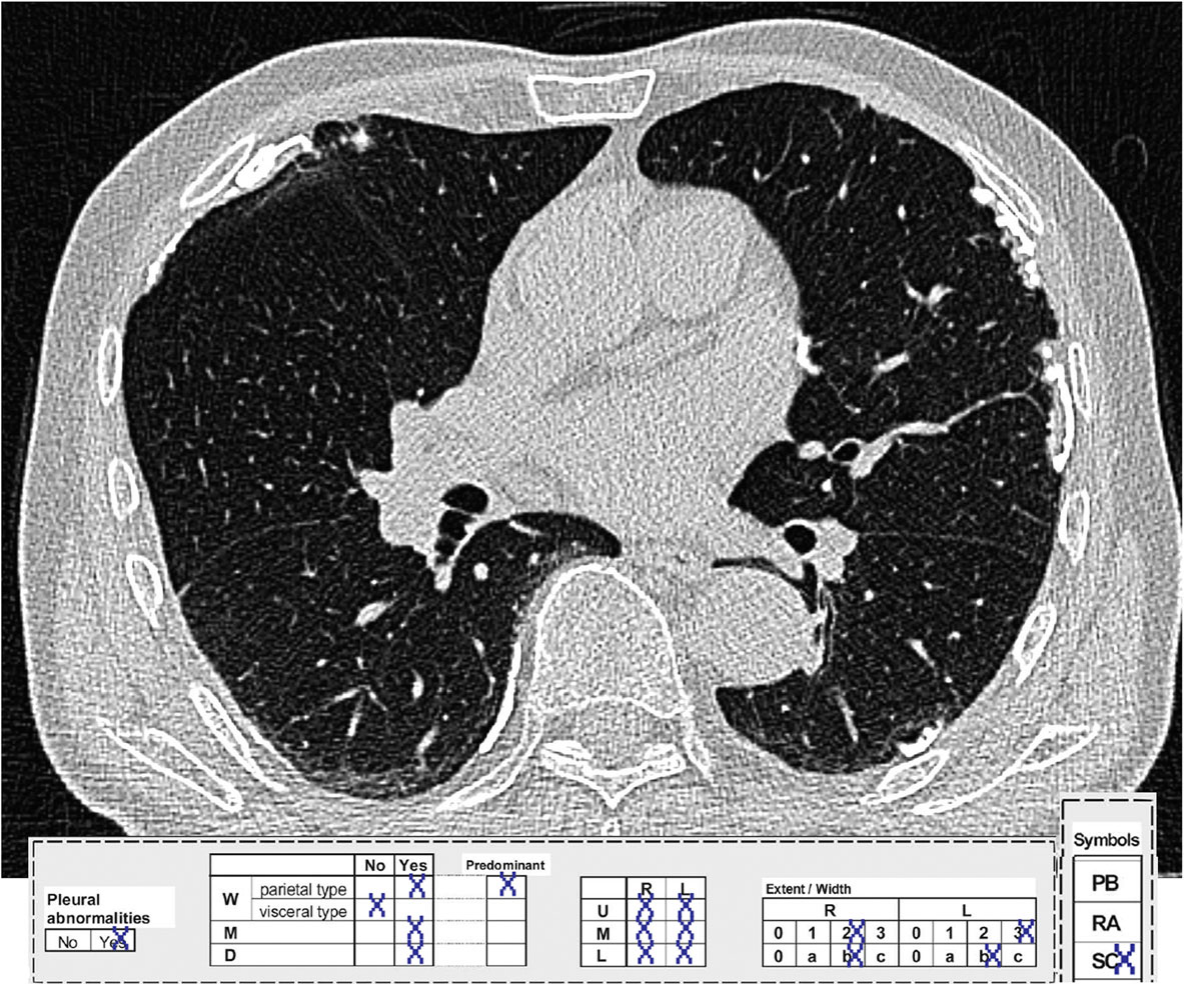
Findings and calculation of score values for pleura plaques and subpleural curvilinear lines in a representative patient: 6 points (for the distribution of pleural plaques) multiplied with 2 points (value from 1 + PB + RA + SC + EF) = 12

In addition to these *scores A* and *B* a newly developed *combined pleural score* is tested. Besides the extent of parietal pleural plaques, typical alterations such as visceral pleural thickening are also considered. These diffuse pleural thickenings are taken into account in this new score by additional point values for ‘parenchymal bands’ (PB), ‘round atelectases’ (RA), ‘subpleural curvilinear lines’ (SC) and ‘effusion, free or loculated pleural fluid’ (EF). The point value from the distribution of pleural plaques (0–6 points), as described above, is now multiplied by the score value from 1 + PB + RA + SC + EF, i.e. 1–5 points. This *combined score* thus allows a value of 0–30. Figure 1 depicts the score calculation for a representative study subject.

Further ICOERD-based scores were tested weighting differently the pleural and pulmonary extensions and additional findings; e.g. a score adding additional points for calcification and the additional attributes PB and RA, a score from the addition of the pleural point values (1–6) and the mentioned extra points with the pulmonary point values (1–18), and a score as addition of the above mentioned score with the score of the parenchymal fibrosis. Since these scores could not demonstrate sufficient predictive value or specificity, the score systems were not presented here.

### Statistical analysis

ICOERD-scores indicating the presence of pleural plaques (corresponding to the above-mentioned score value 0–6) and the lung function parameters vital capacity (VC), diffusion capacity D_LCO_, and transfer coefficient D_LCO_/VA (also called KCO) were included for testing statistical correlation. The lung function parameters were fitted into statistical model in % of the predicted value to compensate for deviations in absolute values due to differences in body size and age [27]. VC, D_LCO_ and D_LCO_/VA as well as the scores of the pleural plaques (*score A)*, the parenchymal fibrosis (*score B)* and the score for pleural plaques with visceral involvement (*combined score*) were tested for correlation.

Since the study group is consisting of less than 50 subjects, the Shapiro-Wilk test was performed to test for normal distribution within the individual groups. The correlation coefficient (Spearmans rho, ρ) and statistical significance were determined to measure the direction and strength of a possible linear relationship between the individual parameters of spirometry, diffusion capacity and CT findings. An ANOVA variance analysis was performed to compare the differently classified groups with each other. Further analyses were carried out using four-field matrix. Therefore individuals were classified as ‘healthy’ or ‘sick’ by exceeding or undercutting the lower limit of normal (LLN) for the respective lung function parameter tested and by the presence of pleural plaques or parenchymal fibrosis in the TCT. The latter is present when at least an ICOERD-score of 2 is reached [5]. Accuracy and Cohens Kappa were calculated.

The program “R” (version 3.5.0) was used for the statistical analyses. Participants gave written (informed) consent to clinical studies.

## Results

The exclusively male patient collective was fairly homogeneous with regard to age, height, weight, BMI and haemoglobin content of the blood (Hb) (Table 1). Of the 41 patients, 10 (24%) reported continuing active smoking, 22 (54%) were former smokers, 9 (22%) had never smoked. The smokers had a cigarette consumption of 25.6 pack years (py) (SD ± 20.8). The exposure duration (in years) to dusts containing asbestos was also determined. There were no statistical correlations to the changes in lung function values. This aspect will therefore not be pursued further.

**Table 1:** Demographic data of the patients (*n* = 41)

Variable	Mean	SD	Median
age [years]	69.8	6.9	72
height [cm]	174.7	6.4	176
weight [kg]	88.9	15.2	88
BMI [kg/m^2^]	29.1	4.5	29.4
Cigarette smoking [py]	25.6	20.8	30
Hb [g/dL]	14.6	1.5	14.6

*SD* standard deviation, *py* pack years, *Hb* haemoglobin

### Spirometry, whole body plethysmography, diffusion capacity (D_LCO_) and transfer coefficient (D_LCO_/VA), and the ICOERD

Table 2 shows the homogeneous distribution for the measured values from spirometry, whole body plethysmography and CO diffusion capacity. The VC shows slight limitations compared to the predicted value (mean 96.47% ± 17.96). The D_LCO_ is also reduced compared to the predicted value (mean 76.35% ± 16.58) while D_LCO_/VA shows minor deviation from the reference value (mean 102.29% ± 21.88).

**Table 2:** Results of spirometry, body plethysmography, D_LCO_ and D_LCO_/VA (*n* = 41)

Variable	Mean	SD	Median	%pred.
VC_max_ [L]	3.93	0.89	4.00	96.47
FVC [L]	3.91	0.91	3.93	95.15
FEV_1_ [L]	2.84	0.79	2.78	90.90
sR [kPa*s]	0.71	0.43	0.62	80.80
RV [L]	2.75	0.64	2.67	105.70
TLC [L]	6.68	1.16	6.57	96.95
RV/TLC [%]	41.35	7.59	39.64	100.38
D_LCO_ [mmol*min^−1^*kPa^− 1^]	6.66	1.48	6.93	76.35
D_LCO_/VA [mmol*min^−1^*kPa^− 1^]	1.30	0.26	1.30	102.29
VIN [L]	3.64	0.89	3.64	86.68

*VC*_*max*_ maximum vital capacity, *FVC* forced expiratory vital capacity, *FEV*_*1*_ expiratory one-second capacity, *sR* specific resistance, *RV* residual volume, *TLC* total lung capacity, *RV/TLC* share of residual volume in total lung capacity. *D*_*LCO*_ haemoglobin value (Hb) corrected diffusion capacity with CO, *D*_*LCO,*_*/V*_*A*_ Hb corrected diffusion capacity with CO relative to alveolar volume, *VIN* inspiratory volume, *SD* standard deviation, *%pred.* % of predicted value

Finally, according to the TCT 27 (66%) of the 41 patients were diagnosed with parietal-type pleural plaques; six (15%) patients showed additional or isolated diffuse pleural thickening; 22 had calcifications in the plaques. The high prevalence of parietal pleural plaques in the study population is caused by and typical for occupational-related asbestos exposures [8]. The score for the localization of the parietal plaques (*score A*) are predominantly 2 points with *n* = 10, only a few have 3 points (*n* = 3) and 4 points (*n* = 4). One patient achieves only one point or the maximum value of 6 points. Additional points for diffuse pleural thickening were given for PB in 14 cases, RA in 2, SC in 8, and EF in 2 cases. Further symbol markings, as specified in the ICOERD, serve to identify comorbidities and were only taken into account in this evaluation if they indicated the presence of exclusion criteria, e.g. heart disease. Eighteen patients show pulmonary fibrosis, only one patient honey combing.

**Fig. 2:**
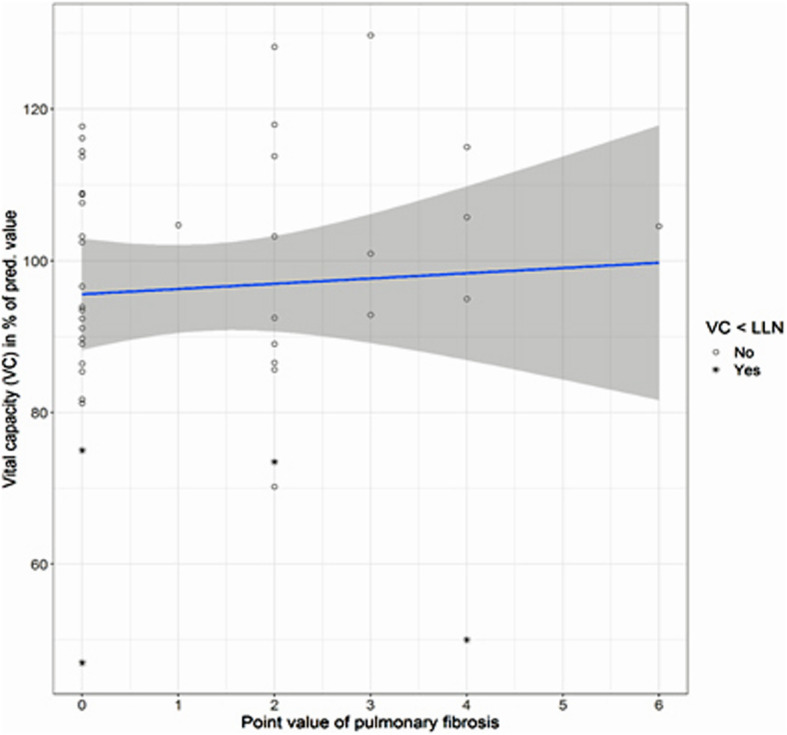
Comparison of VC with the *score B* of pulmonary fibrosis according to ICOERD coding scheme (*n* = 41). *ρ* = 0.10; n.s. The pathological values of the VC (< lower limit of normal (LLN)) are marked with *

**Fig. 3:**
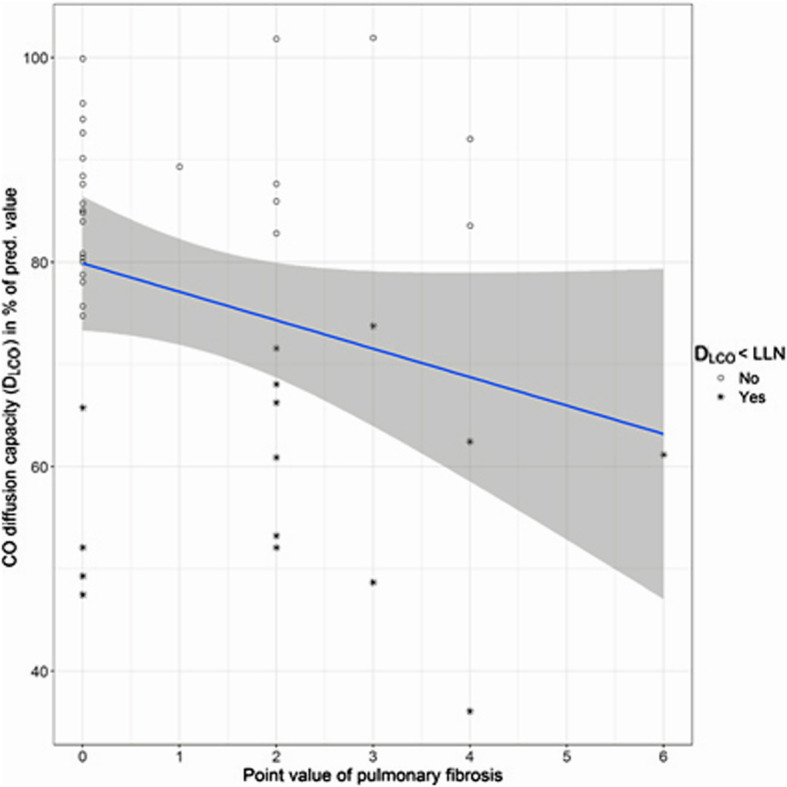
Comparison of diffusion capacity with the *score B* of pulmonary fibrosis according to ICOERD coding scheme (*n* = 41). *ρ* = − 0.22; n.s. The pathological values of the D_LCO_ (< lower limit of normal (LLN)) are marked with *

**Fig. 4:**
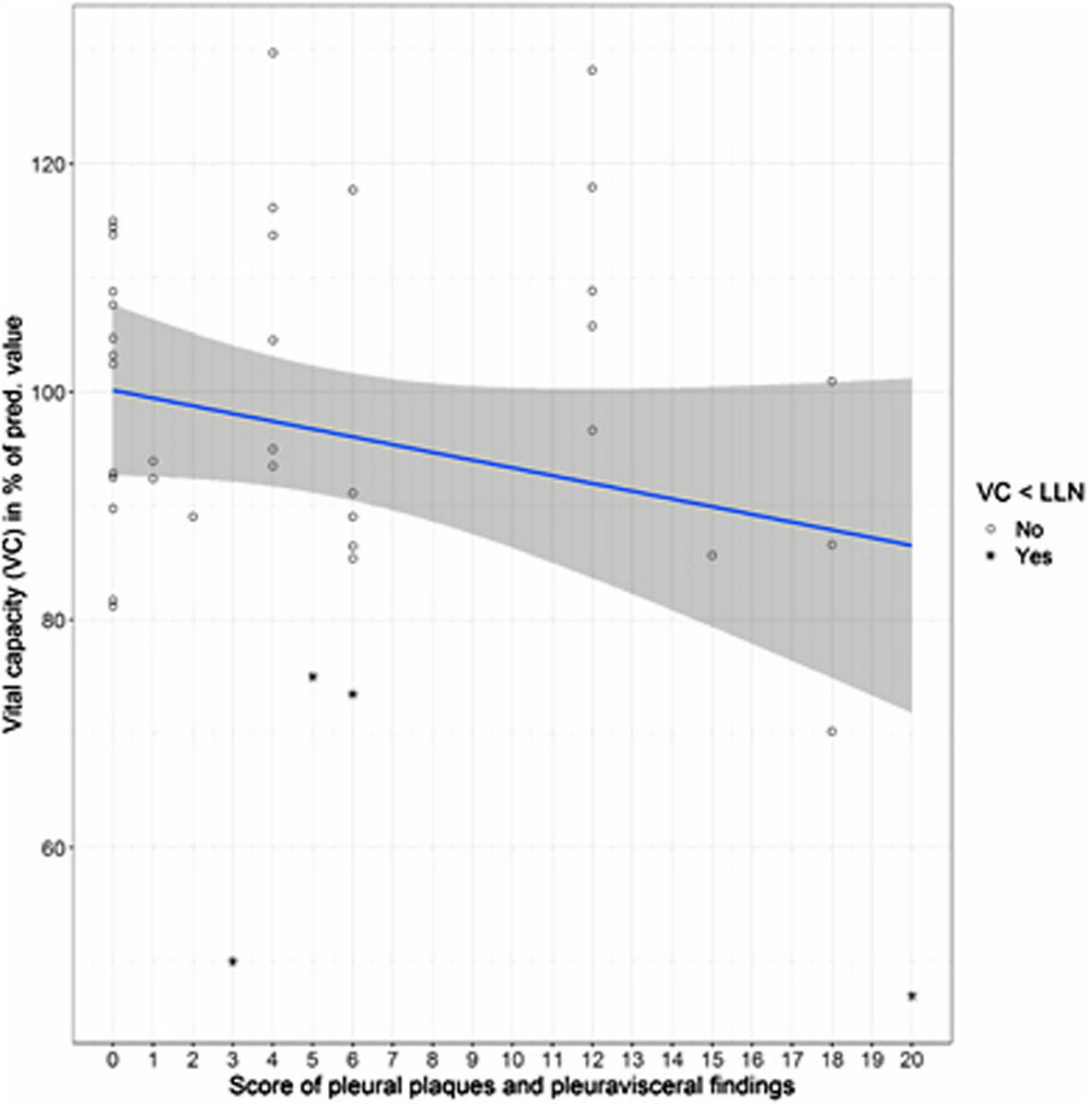
Comparison of vital capacity with the *combined score* taking into account parietal and visceral pleural alterations (*n* = 41). *ρ* = − 0.18; n.s. The pathological values of the VC (< lower limit of normal (LLN)) are marked with *

**Fig. 5:**
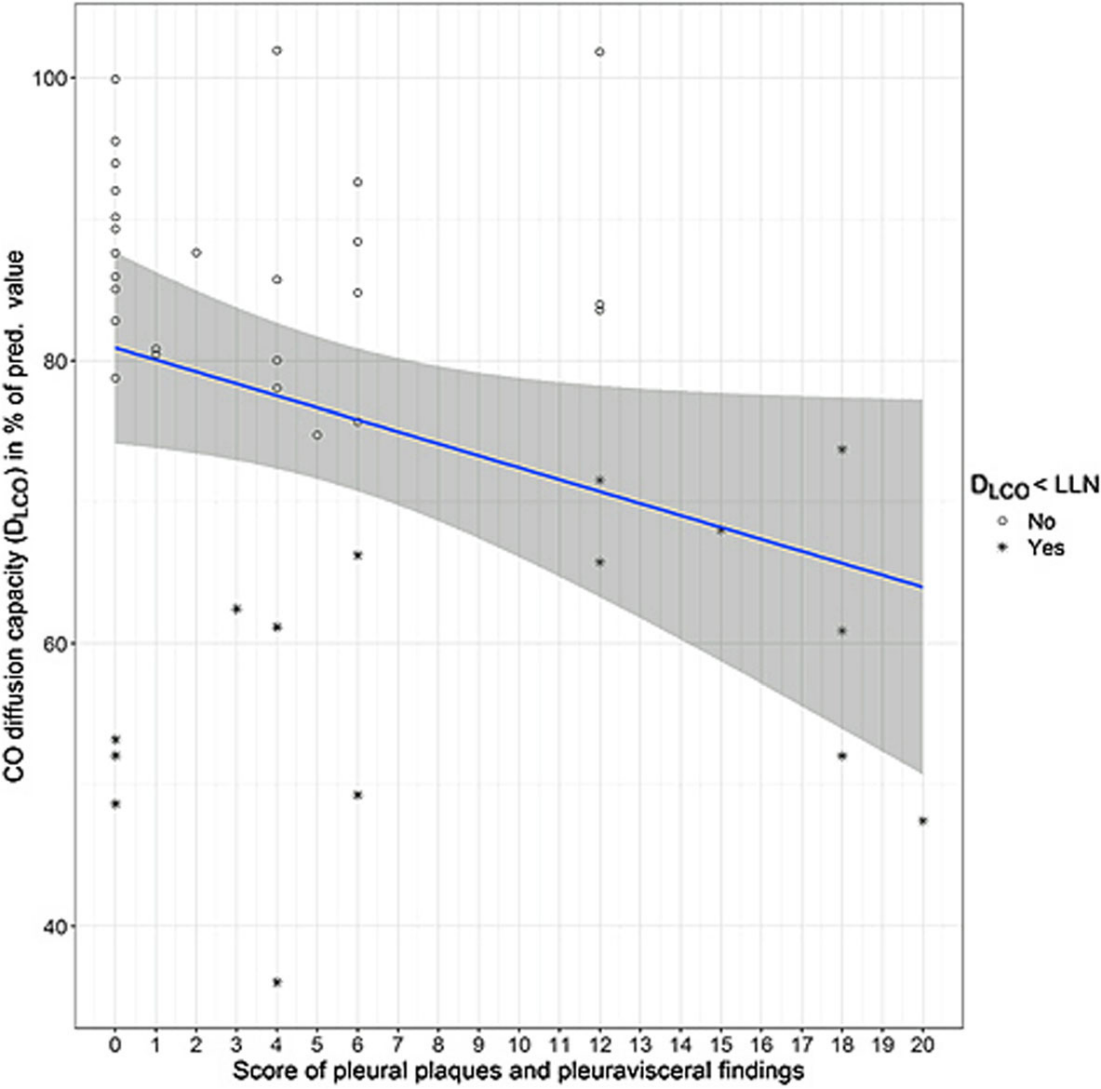
Comparison of diffusion capacity with the *combined score* taking into account parietal and visceral pleural alterations (*n* = 41). *ρ* = − 0.35; *p* = 0.03. The pathological values of the D_LCO_ (< lower limit of normal (LLN)) are marked with *

Figures 2, 3, 4 and 5 show the distributions of the *score B* for parenchymal changes and the newly formed *combined  score* for parietal pleural plaque expansion and diffuse pleural thickening, taking into account PB, RA, SC, and EF in correlation to VC and D_LCO_.

### Comparison of the lung function parameters VC, D_LCO_ and D_LCO_/VA with the presence of pleural plaques in CT

Patients with pleural plaques (*n* = 27) and a score of at least 1 in the evaluation of plaque distribution in the ICOERD coding scheme *(score A)* have a slightly lower vital capacity (VC) compared to patients without pleural plaques (*ρ* = − 0.19, n.s.) (Table 3). Likewise, the mean values of the VC in the separate evaluation of both groups indicate that patients of the collective without pleural plaques in CT show a higher VC on average than patients with pleural plaques (100.1 and 94.6%, respectively; Table 3). The sensitivity of the VC is correspondingly low in the study group, only 4 of 27 (15%) patients with one or more pleural plaques have pathological VC value (NPV 38%; Table 4). In contrast, all patients with a significant reduction of VC (<LLN) also have pleural plaques in CT findings (PPV 100%). The Shapiro-Wilk test shows a normal distribution for VC as well as for D_LCO_ and D_LCO_/VA (Table 3).

**Table 3:** Comparison of vital capacity (VC), diffusion capacity (D_LCO_), and transfer coefficient (D_LCO_/VA) of subjects without and with pleural plaques or without and with pulmonary fibrosis in TCT

Shapiro-Wilk-Test			VC [% pred.]		D_**LCO**_ [% pred.]		D_**LCO**_/VA [% pred.]	
			*p* = 0.15		*p* = 0.06		*p* = 0.40	
		**n**	Mean	SD	Mean	SD	Mean	SD
**Pleural plaques**	none	14	100.1	11.6	79.7	16.1	98.0	23.1
	yes	27	94.6	20.5	74.6	16.8	104.5	21.3
correlation ρ of pleural plaques (*score A*) with lung function parameters			−0.12		−0.25		0.12	
		**n**	**VC** [% pred.]		**D**_**LCO**_ [% pred.]		**D**_**LCO**_**/VA** [% pred.]	
			Mean	SD	Mean	SD	Mean	SD
**Pulmonary fibrosis**	none	23	95.7	16.2	80.0	14.2	106.2	20.6
	yes	18	97.5	20.4	61.3	18.0	97.3	23.6
correlation ρ of pulmonary fibrosis (*score B*) with lung function parameters			0.10		−0.22		−0.26	
**Combined score**								
correlation ρ of the *combined score* of parietal pleural changes and diffuse pleural thickening with lung function parameters			−0.18		− 0.35*		0.09	

*ρ* correlation (Spearmans rho, *LLN* lower limit of normal; * significant (*p* < 0.05)

**Table 4:** Four-field matrixes for comparison of lung function parameters with findings of pleural plaque^a^ resp. of pulmonary fibrosis^b^

CT		VC		D_**LCO**_		D_**LCO**_/VA	
		≥ LLN	< LLN	≥ LLN	< LLN	≥ LLN	< LLN
**Pleural plaques**	none	14	0	11	3	11	3
	yes	23	4	15	12	26	1
Accuracy		43.9%		56.1%		29.3%	
Cohens Kappa		0.10		0.19		−0.13	
**Pulmonary fibrosis**	none	21	2	19	4	22	1
	yes	16	2	7	11	15	3
Accuracy		56.1%		73.2%		61.0%	
Cohens Kappa		0.03		0.45		0.13	

^a^pleural plaque means ≥1 P. in *score A*; ^b^pulmonary fibrosis means ≥2 P. in *score B*; *VC* vital capacity, *LLN* lower limit of normal, *D*_*LCO*_ diffusion capacity with CO, *D*_*LCO*_*/VA* diffusion capacity with CO in relation to alveolar volume

Considering the correlation between the score value for pleural plaques and the evidence of a pathological CO diffusion capacity, similar to the result for VC, only a weak negative correlation is found (*ρ* = − 0.19, n.s.; Table 3).

In the four-field matrix, the D_LCO_ of patients without plaques is on average 79.7%, i.e. already in the lower limit range of the predicted values and lower than the VC (Table 3). In the individual analysis, however, only 3 out of 14, i.e. 21% of these patients without plaques, show a reduction in D_LCO_ below LLN, whereas 44% (12 out of 27) of patients with at least one pleural plaque show D_LCO_ below LLN (Table 4). With 56.1% accuracy, the D_LCO_ thus gives a better indication of the presence of pleural plaques than the vital capacity (accuracy 43.9%). Thus the D_LCO_ shows a positive predictive value of 80% (specificity 79%, sensitivity 44%). Cohens Kappa just indicated a slight correlation of the expected with the observed accuracy for VC; for the D_LCO_ a nearly sufficient correlation is shown (0.10 and 0.19 resp.; according to [32]).

The transfer coefficient D_LCO_/VA shows a pathological value in only four patients; the accuracy for the presence of pleural plaques is low with 29.3% (Table 4). The mean values of this measurement are close to the predicted value in all subgroups; asbestos-related lung and pleural changes do not lead to a reduction of D_LCO_/VA in this collective, the correlation between pleural plaques and D_LCO_/VA is poor (Cohens Kappa − 0.13) (Tables 3 and 4).

### Comparison of lung function parameters VC, D_LCO_ and D_LCO_/VA with the detection of pulmonary fibrosis

Pulmonary, i.e. parenchymal, fibrosis is present if at least 2 fields have been marked in the ICOERD coding scheme [5], corresponding to a *score B* ≥ 2; higher values indicate a greater extent of pulmonary fibrosis. As mentioned above, 18 patients show pulmonary fibrosis with a *score B* of 1–6 (of max. 18) points. The vital capacity shows no and the D_LCO_ a moderate correlation to the extent of pulmonary fibrosis (*ρ* = 0.10 and − 0.22, resp., n.s.; Table 3). The relation between the score, i.e. the extent of pulmonary fibrosis, and the pulmonary function parameters VC and D_LCO_ are visualized in Figs 2 and 3.

In patients with pulmonary fibrosis and pathological lung function values, the D_LCO_ shows the highest accuracy with 73.2%, followed by the VC with an accuracy of 56.1% (see four-field matrix, Table 4). Cohens Kappa of 0.45 shows moderate correlation between D_LCO_ and pulmonary fibrosis. Once again, D_LCO_/VA shows no association to the radiological findings with regard to pulmonary fibrosis (Tables 3 and 4).

### Comparison of lung function parameters VC, D_LCO_ and D_LCO_/VA with the score of parietal pleural changes and diffuse pleural thickening

This newly developed score considers the local extent of pleural plaques and visceral and subpleural asbestos-related alterations corresponding to the presence of PB, RA, SC and EF as described above. The score values range between 0 and 20. For the vital capacity at least a weak correlation with this score is apparent; the D_LCO_ shows a mean significant correlation with the score (*ρ* = − 0.35, *p* < 0.05; Table 3). These correlations of lung function parameters and scores are illustrated in Figs 4 and 5. The D_LCO_/VA shows no correlation to this score (Table 3). The four-field matrix is not suitable for this score; it would show the same results as for the presence of pleural plaques.

In addition, we have calculated a further score from the addition of the *combined score* (pleural changes with visceral changes) with the *score B* for the extent of pulmonary fibrosis. This allows both pleural changes and lung changes caused by asbestos to be adequately considered. The comparison of this score with the D_LCO_ showed the highest correlation (*ρ* = − 0.41; *p* < 0.01) and the VC shows only a weak correlation (*ρ* = − 0.12; n.s.) (Data are not shown in figures).

## Discussion

Lung function testing is a simple, non-invasive and inexpensive diagnostic method for early detection and verification of a disease or its progression, thus enabling early intervention. TCT classified by ICOERD shows the extent of asbestos-related morphological changes, but not the effects on volume and gas exchange in the lungs. The aim of this study was to determine whether the results of pulmonary function tests (spirometry and D_LCO_) can increase the predictive value for the presence of asbestos-related fibrosis.

The classification of the visceral type of asbestos-related pleural fibrosis with the ICOERD coding scheme [29] requires the specification of at least one finding in the category “symbols” listing intrapulmonary changes like ‘parenchymal bands’ (PB) and ‘round atelectases’ (RA). Diagnostic findings as ‘subpleural curvilinear lines’ (SC) and ‘effusion, free or loculated pleural fluid’ (EF) are associated with previous exposures to asbestos and have therefore been included in our newly developed scoring system.

According to our pathophysiological model, besides PB, RA, SC, and EF, other diagnostic findings listed in the ICOERD coding scheme could also affect the functionality of the lung, such as calcification of the plaques. For this purpose, the *combined score* included these diagnostic findings and was applied within the study collective. The consideration of PB and RA additionally to the pleural plaque values, but without the scores for SC and EF was also tested. By including PB, RA, SC, and EF in addition to the extent of pleural plaques in the statistical model, significant changes in lung function could be demonstrated.

Compared to spirometry with VC determination, our study showed D_LCO_ to be the more sensitive diagnostic method with better accuracy for detecting asbestos-related changes in the lung and pleura. With the high positive predictive value of 80% and a specificity of 79% reduction of the D_LCO_ is strongly associated with radiological findings (CT) of asbestosis.

Vital capacity also showed a negative correlation, when the pleural plaques were taken into account. In our statistical analysis there was no dependence of VC on the severity of fibrotic parenchymal changes. This might be attributed to the moderate fibrosis (maximum score was 6 of theoretically achievable 18 points) in all cases. Likewise, Şener et al. [33] described only a weak negative correlation between FEV_1_ and FVC decrease with the increase of small opacitiy grades in HRCT; however, the D_LCO_ was not investigated by these authors. Barnikel et al. [34] showed a decrease of FVC and D_LCO_ in 56 asbestos exposed subjects, particularly significant in cases with fibrotic phenotype in HRCT. The present study confirms the results of Park et al. [15] and Miles et al. [35] who described the decrease of D_LCO_ in the presence of asbestos-related changes depending on the extent of pleural plaques, diffuse pleural thickening and asbestosis. In addition, Cha et al. [16] found a decrease in D_LCO_ in the presence of manifest asbestos-related pleural plaques although not statistically significant; however, this study did not consider additional abnormalities like PB or SC in their analyses.

In the present study, D_LCO_/VA was not sensitive, confirming the results of van der Lee et al. [36] and Hughes & Pride [37] where the transmission coefficient D_LCO_/VA had no diagnostic added value compared to the parameters D_LCO_ and VC for the detection of diffuse parenchymal diseases. In accordance, Schikowsky et al. [38] showed no impairment of D_LCO_/VA and no reduction in VC in subjects formerly exposed to asbestos. In difference, the D_LCO_ results were not presented.

One of the strengths of the present study is the limitation to study participants without confounding factors. This applies in particular to the exclusion of patients with heart disease and emphysema, as the latter can be caused by inhalation of mineral dusts, but also by smoking. Both diseases can significantly impair the results of lung function, especially the D_LCO_. The results can be regarded as specific for the changes caused by asbestos.

We can show no dependency between lung function and the dose of previous exposure to asbestos. This may be due to inaccuracies in the recording of these events, which date back decades. Often, only rough dose-effect relationships are described for the development of asbestos-related changes [39]. All study participants provided explicit proof of occupational exposure to asbestos; therefore all were diagnosed with asbestosis, not as interstitial pneumonia or idiopathic pulmonary fibrosis, as even the small amounts can lead to the occurrence of asbestosis [8, 39, 40].

### Limitations

Because the patient data were collected over a period of 4 years, a particular challenge arose from the fact that different CT scanners had been used, which had to meet certain quality criteria depending on the CT scanner. After careful review, data sets that did not meet the defined CT quality criteria had to be removed from the study [8].

Exposure to asbestos fibre dust in combination with smoking has significant effects on the findings both in TCT and in lung function tests [41]. Especially in heavy smokers, these findings can be difficult to distinguish from patients with mild asbestosis. However, these were only a few subjects (Table 1) and we did not analyse them separately.

The assessment of the TCT after ICOERD was performed by radiologists familiar with pneumoconiosis; however, no controls could be performed by a second radiologist. Minor inaccuracies in the calculated scores could therefore occur, even if the interreader variability in the findings with the ICOERD is low, as Suganuma et al. [30] showed.

TCT and pulmonary function were usually not performed on the same day, in some cases the tests were several months apart. This had to be accepted due to the very slow progression of asbestos-related fibrosis, as conducting such a study does not allow an indication for further radiation exposure to TCT.

## Conclusions

The correlation of lung function changes and TCT findings was strongest when D_LCO_ was used and ICOERD classification of TCT included parenchymal bands, subpleural curvilinear lines, round atelectasis, and pleural effusion in addition to pleural plaque extension. In correlation to the exclusively pulmonary findings, D_LCO_ showed the highest accuracy, but VC only a weaker correlation. Therefore, lung function parameter D_LCO_ should be included as complementary parameter to VC in future examinations for the detection of asbestosis. In the evaluation of radiological imaging of morphological asbestos-related lung and pleural changes, the presence of parenchymal bands, subpleural curvilinear lines, round atelectasis and pleural effusion are predictive for pulmonary insufficiency and might also be included in the evaluation of ICOERD-findings.

## Data Availability

Not applicable.

## Abbreviations

%pred.: Percent of predicted value
ANOVA: Analysis of variance
ATS: American Thoracic Society
BMI: Body Mass Index
CO: Carbon monoxide
Cohens κ: Cohen’s kappa coefficient
D_LCO_: CO diffusion capacity
D_LCO_/VA: Diffusion capacity with CO in relation to alveolar volume
ECSC: European Coal and Steel Community
EF: Effusion, free or loculated pleural fluid
ERS: European Respiratory Society
FEV_1_: Forced expiratory vital capacity over one second
FEV_1_/FVC: Lung volumes at normal relative one-second capacity
FVC: Forced expiratory vital capacity
GLI: Global Lung Initiative
Hb: Haemoglobin content of the blood
HRCT: High-resolution computed tomography
ICOERD: Computed Tomography for Occupational and Environmental Respiratory Diseases
L: Left
L: Lower field
LLN: Lower limit of normal
M: Middle field
Mean: Average (mean) value in a sample
Median: Median value of a range of values
n: Number (of subjects included)
n.s.: Not significant
*p*: Probability value
PB: Parenchymal bands
py: Pack years
R: Right
RA: Round atelectasis
resp.: Respective
RV: Residual volume
RV/TLC: Share of residual volume in total lung capacity
SB: Single breath
SC: Subpleural curvilinear lines
SD: Standard deviation
sR: Specific resistance
TCT: Computed tomography of the thorax
TLC: Total lung capacity
U: Upper field
ULN: Upper limit of normal
VC: Vital capacity
VC_max_: Largest measured vital capacity
VIN: Inspiratory volume
ρ: Spearman’s rank correlation coefficient (Spearman’s rho)
